# Emerging nursing scholars guide to peer reviewing an academic manuscript

**DOI:** 10.1002/nop2.368

**Published:** 2019-08-28

**Authors:** Sarah Oerther, Roger Watson

Conducting a peer review for a journal may help emerging nursing scholars grow in their understanding of the publication process. Peer review, or the use of peers or experts to assist in judging the value of submitted work, is used—in common with other fields—in nursing to help decide which manuscripts are published in nursing journals and how they should be changed before publication (Godlee & Jefferson, 2003; Polit & Beck, 2017). The process of peer review in nursing research may have a significant impact on what scientific information goes into the public domain. In nursing science, this information can have a major influence on what happens to patients. As nursing science become more multifaceted, the role of peer review has become more prominent (Godlee & Jefferson, 2003; Polit & Beck, 2017).

Unfortunately, there is almost no formal or standardized training for emerging nursing scholars as they begin to engage in the peer‐review process. Most publishers do, however, offer advice (e.g., Pierson, undated; Wiley, 2019). The purpose of this editorial is fivefold: first, to give suggestions of how emerging scholars can become reviewers; second, to give emerging nursing scholars a general overview of how to conduct a peer review for a nursing journal; third, to give questions to address in the review; fourth to give practical tips for how to give feedback to editors; and finally, to give practical tips for how to give feedback to authors.

## 1 HOW EMERGING SCHOLARS CAN BECOME REVIEWERS

Many journal editors encourage emerging scholars to become reviewers. A great place to start is by identifying a nursing journal you would like to review a manuscript. Visit the home page of the journal, find the editor and send the editor an email offering to review a manuscript. If you do become a reviewer, ensure that you register with Publons (https://publons.com/about/home/; accessed 1 August 2019) as this will ensure—for journals that are also registered with Publons—that your reviewing activity is recorded and does not go unrecognized (Watson, 2018).

Table 1 provides an essential glossary of terms often used by journal editors.

**Table 1:** Essential terms used by editors of journals

Term	Definition
Clarivate impact factor	In a particular year, this is the measure of the frequency that an ‘average article’ published in the previous two years in a journal has been cited.
DOAJ Seal	DOAJ stands for Directory of Open Access Journals. The seal is a certification for open access journals that have high publishing standards, achieve a high level of openness, and adhere to best practices.
Editorial Board	This is a Board that consists of selected, unpaid experts in the academic field covered by the journal.
Editor‐in‐chief	This is the editorial leader who has final responsibility for a journal's policies and operations.
Orcid ID	The ORCID is a non‐proprietary alphanumeric code to uniquely identify scientific and other academic authors and contributors.
Publons	This is a website that verifies, tracks, and showcases academics’ editorial and peer review contributions for academic journals (for free).
Refereeing	This is another term used for scholarly peer review.
ScholarOne	ScholarOne is a website that manages the workflow for some academic journals.

## 2 GETTING STARTED

Journals also reach out to emerging scholars. Generally, if a journal considers you as a prospective peer reviewer, you will receive an email from the journal. However, first make sure this is not a predatory journal. If you are unfamiliar with the concept of predatory journals, then these are journals of very low quality, with very low standards of quality assurance and normally they merely want to make use of your name rather that to engage you in a valid peer‐review process. They are relatively easily spotted as they almost invariably use the inane ‘Greetings!!’, at the start of the email, make some statement to indicate that they hope you are having a ‘great day’ and then pay you some obsequious compliment. They also masquerade under names that are very general and inclusive and often like established, reputable journals (e.g., *Advanced Nursing Journal* to sound like *Journal of Advanced Nursing*). The problem of predatory journal has been addressed by one of us (RW) on several occasions (Pickler et al., 2014; Watson, 2017a, 2017b, 2018, 2019).

Within the email invitation to review an article, you most likely will be sent a link to the abstract of the manuscript to help you decide if you are qualified to do the review. For emerging nursing scholars, this is likely to be a very tempting opportunity. We recommend you make sure you have time to conduct the review (i.e., at least an hour) and that you make sure you are familiar enough with the content or methods to produce a quality review (i.e., have completed formal training or conducted research using methods in the same or a highly related area) (Godlee & Jefferson, 2003; Polit & Beck, 2017). Also, ask yourself if you have any conflict of interest (Wiley, 2019). If there are any concerns, contact the editor of the journal to discuss your doubts and obtain advice.

You should also be clear what type of review process the journal operates, as this can vary (Ali & Watson, 2016). Most nursing journals use double‐blind peer review whereby the author and the reviewer remain unknown to each other during the review process. While, reviewers may subsequently identify the authors of published articles they have reviewed, the reviewer remains permanently anonymous. But some journals use open peer review, and this is becoming more common. Both parties are known to the other, and moreover, the reviews are published alongside the published article. This is not the place to debate the virtues of these systems of peer review but as a novice reviewer you should ensure that you are happy to have your identity known by the authors if you are asked to participate in open peer review.

### 2.1 First quick read‐through

If you accept the invitation to review, we suggest you start the review by doing a quick read‐through of the entire manuscript without judgement. A quick read‐through should give you an understanding of the purpose, key results and conclusion of the manuscript and give you a sense of whether you will eventually suggest that the editor accept or reject the manuscript (Godlee & Jefferson, 2003; Polit & Beck, 2017; Wiley, 2019).

A quick read‐through might also save you time by flagging fatal flaws early in your review process. Fatal flaws may include the use of a method that has been discredited, drawing conclusions that are contradicted by the author's own statistical or qualitative evidence or maybe overlooking a method that is known to influence this area of nursing research (Godlee & Jefferson, 2003; Polit & Beck, 2017; Wiley, 2019). You may also decide whether the manuscript makes a significant contribution to the field. The information in the manuscript may already be well established but it is also the case that replication of studies is considered necessary and laudable. It is for you to judge as the process of peer reviewing is not scientific. For example, yet another study indicating that adolescents do not like having type I diabetes or that sexual function may be adversely affected by stroke is probably not necessary; these things are well known. However, a large study replicating a previous intervention study—whether by the same or a different team—will add to the database for subsequent meta‐analysis. You must ensure, of course, that the authors are clear that it is a replication and not claiming it as original study.

Nursing research should be replicable and robust (Godlee & Jefferson, 2003; Polit & Beck, 2017; Wiley, 2019). If an article is suffering a fatal flaw, it is important to provide clear evidence to the editor in your review.

### 2.2 Second detailed read‐though

Once you have completed your first read and you have decided the manuscript may be publishable, the purpose of the second detailed read‐through is to help prepare the manuscript for publication (Wiley, 2019). Take notes because this will help you write a review that summaries your comments about the manuscript. Remember, as a reviewer; your comments should accomplish two outcomes, namely (a) providing evidence for the editor to decide with respect to accepting or rejecting the work and (b) providing suggestions to the author of improvements to increase readability, clarity or quality of their work. Also, you will want to provide clear suggestions for ways the author can address the problems you have raised (Godlee & Jefferson, 2003; Polit & Beck, 2017; Wiley, 2019). As a reviewer, if you are going to raise a concern, suggest improvement.

## 3 QUESTIONS TO CONSIDER WHEN WRITING A REVIEW

### 3.1 Introduction

The authors of a manuscript should use the introduction to state the main question addressed and summarize the goals, approaches and the conclusion of the paper. As a reviewer, it is your job to determine if the authors have contextualized the research (Godlee & Jefferson, 2003; Polit & Beck, 2017; Wiley, 2019). Some questions to ask while doing your second read‐through include:

- Did the authors give a clear idea of the target readership and why the research was carried out?
- Was the problem easy to identify?
- Did the research question match the method used?
- Does the author's research contribute to any knowledge that is useful to the nursing discipline?

The following is an example of how you might summarize your feedback to authors:

The authors did try to use the introduction to contextualize the problem in relation to published research/reports, stating the importance of (insert topic). I was able to quickly identify the problem statement and I can understand how this problem is related to nursing. I had trouble understanding the variables involved in this study. Perhaps a better definition of (insert the variables) could be included in the opening paragraph. This could aid in understanding these concepts prior to reading the rest of the paper.

### 3.2 Background/literature review

The authors of a manuscript should use the background or literature review to synthesize relevant literature and to describe why the study is needed. As a reviewer, it is your job to determine if the authors’ use of previous research is related to the study. Most of the time, the background should end with a research question (Godlee & Jefferson, 2003; Polit & Beck, 2017; Wiley, 2019). Some questions to ask while doing your second read‐through include:

- Was the literature review up‐to‐date and based mainly on primary sources?
- Did the authors provide state‐of‐the‐art synthesis of the literature? Alternatively, did the authors describe a few previous studies and forgo synthesis?
- Did the authors provide a strong rationale for the study?
- Did the authors highlight gaps in current understanding or conflicts in current knowledge?
- Did the authors define key concepts adequately?
- Was a conceptual/theoretical framework articulated? If not, is the absence justified?

The following is an example of how you might summarize your feedback to authors:

The background section provides lots of detail on (insert topic). Consider summarizing the detail a little more concisely and connecting it to (insert topic). The gaps in current knowledge need to be more clear and the author needs to define key variables.

### 3.3 Research design and methodology

The authors of a manuscript should use the design section to provide a clear statement of the design. Then, they should use the method section to describe the methods used to carry out the design, the psychometric properties of any questionnaires and, if the study is quantitative, a power analysis should be stated. The authors should also describe randomizing, blinding and control, or approaches to rigor in qualitative studies. As a reviewer, it is your job to determine if the authors’ analysis of the data includes enough detail for qualitative or quantitative analysis for the process to be clear to readers (Godlee & Jefferson, 2003; Polit & Beck, 2017; Wiley, 2019). Some questions to ask while doing your second read‐through include:

- Did the authors use appropriate procedures used to safeguard the rights of study participants?
- Was the study externally reviewed and approved by an IRB/ethics review board?
- If the study involved an intervention with human participants, was it appropriately prospectively registered in accord with AllTrials principles or WHO requirements (Noyes, 2018)?
- Were the data collection points appropriate?
- Did the design minimize biases and threats to the validity of the study?
- Was the population described in adequate detail?
- Did the authors try to minimize sampling biases?
- Was the sample size based on a power analysis?
- Was there congruency between the operational and conceptual definitions?
- Did the authors use appropriate methods to measure key variables?
- Did the authors adequately described specific instruments, and were they good choices, given the study population and the variables being studied?
- Did the authors provide evidence that the data collection methods yielded data that were reliable and valid?
- Did the authors provide evidence that the staff who collected the data were adequately trained?

The following is an example of how you might summarize your feedback to authors:

The authors did match the methods to the research question. The authors need to discuss the validity and reliability of the scales that were used.

### 3.4 Results

The authors of a manuscript should use the results section to tell a coherent story. As a reviewer, it is your job to determine if the authors have clearly described what the data showed and make sure they appropriately referenced the statistical analysis (Godlee & Jefferson, 2003; Polit & Beck, 2017; Wiley, 2019). Some questions to ask while doing your second read‐through include:

- What was the outcome of the analysis?
- What was confirmed or discovered?
- Were the findings adequately summarized, with good use of tables and figures?

The following is an example of how you might summarize your feedback to authors:

In the results section, the authors did not provide an overview of the sample. This section could be strengthened with a description of the actual sample that was studied.

### 3.5 Discussion

The authors of a manuscript should use the discussion section to describe and discuss the overall story that has been created. As a reviewer, it is your job to determine if the authors evaluated the trends observed and explained the significance of the results in relation to a broader understanding of current literature. The discussion section can only be done correctly by referencing published research. If there are gaps or inconsistencies in the story, the authors should address these and suggest ways future research might confirm the finding or take the research forward (Godlee & Jefferson, 2003; Polit & Beck, 2017; Wiley, 2019). Some questions to ask while doing your second read‐through include:

- Was the issue of clinical significance discussed?
- How generalizable are the findings?
- Did the researchers discuss the implications of the study for clinical practice or further research?

The following is an example of how you might summarize your feedback to authors:

In the discussion section, the authors explained how the findings were consistent or inconsistent with the outside literature. Consider summarizing the detail a little more concisely and explain if the findings are generalizable.

### 3.6 Conclusion

The authors of a manuscript should use the conclusion to reflect on the purpose and specific aims, whether they were achieved or not. As a reviewer, it is your job to determine this section is surprising (Godlee & Jefferson, 2003; Polit & Beck, 2017; Wiley, 2019). Some questions to ask include while doing your second read‐through include:

- Is new information being presented?
- Provide recommendations for practice, education or research.

The following is an example of how you might summarize your feedback to authors:

In the conclusion section, the authors presented new terms that had not been discussed previously. Please consider addressing these terms in the background sections. Additionally, this conclusion would be stronger if you provided a few clinical recommendations.

## 4 CONFIDENTIAL FEEDBACK FOR THE EDITOR

Most journals have the option to provide confidential comments to the editor. You are quite entitled to say ‘None’ here if you have nothing to emphasize or any concerns to raise. However, you may wish to emphasize why you are recommending acceptance, so provide a justification of details why, and if any areas could be improved. Where improvement is needed, a recommendation for major or minor revision is typical. If recommending rejection or major revision, you may wish to emphasize this clearly to the editor and provide the editor with a justification of why you are rejecting the manuscript (Wiley, 2019). Especially, you may wish to use the confidential comments to the editor to raise any concerns that you felt unable to raise with the author.

The following is an example of how you might summarize your feedback to an editor:

While I have raised relatively few points regarding the contents of the manuscript with the authors, I am concerned that I think I can identify the research team and they have been the subject of several retracted articles recently. I may be wrong, but I would be very grateful of you could investigate this as it may have some bearing on your decision to proceed.

## 5 FEEDBACK FOR THE AUTHOR

We recommend as a reviewer you provide constructive feedback to authors. It is important to remember that most authors spend a substantial amount of time planning and conducting research before they invest even additional time writing and revising their manuscript before submitting it to a journal. First, as a reviewer, you should provide feedback that respects the investment of time, talent and treasure by the authors (investing four hours to review a paper does not justify the use of unprofessional behaviour when evaluating months or years of work by a peer). Begin the review by summarize what the paper is about including the chief finding. Place the finding of the paper in the context of the existing literature. Indicate the significance of the work and if it is novel or mainly confirmatory. Indicate the work's strengths, its quality and completeness. Then state any significant flaws or weaknesses and note any special considerations. For instance, if previously held theories are being overlooked, give constructive feedback describing the way that they could improve the research. Keep the focus on the research, not the author. Remember to provide constructive criticism in order to help the authors improve, even if recommending rejection. This helps developing researchers improve their work (Godlee & Jefferson, 2003; Polit & Beck, 2017; Wiley, 2019).

For instance, you may start your peer review to the authors by saying:

Thank you for the opportunity to offer feedback. I enjoyed learning more about (insert the topic of the manuscript). I was unable to find a much research on your topic, so I think this paper is worth pursuing. It is obvious to me that you have done lots of work on this paper. It is quite an undertaking to pull together all the necessary information for a research study. Below I have offered comments. I hope you find my comments helpful.

It is then very helpful if you can list your suggestions for change possible numbered or bullet‐pointed and as discretely as possible. This helps the editor and the author to see precisely what needs to be done, and it also helps the author to respond in an organized way as recommended by Williams (2004).

## 6 A FEW THINGS NOT TO DO

Try to be consistent across all aspects of your review. Especially, ensure that your comments are congruent with your recommendation. For example, do not get into the habit of writing copious comments condemning a manuscript and then recommending revision or, *vice versa*, writing a glowing report on a manuscript and recommending rejection. Likewise, avoid writing a glowing report about an article to the author and then raising extreme doubts about the article with the editor and recommending rejection. Never be insulting to authors; try to maintain a scholarly tone in your reviews and convey any criticism, however serious, in polite terms. It is also bad reviewing practice to ask for copious amendments to a manuscript and then to decline to review the revisions. This makes life very hard for the editor and the authors. Finally, editors do not like to be asked, rudely, why they have sent someone a manuscript when it is clearly poor and having their judgement questioned. If you do not want to review a manuscript, then simply and politely decline to review it.

## 7 CONCLUSION

After you submit your review to a journal, some journals may send you the comments made by other peer reviewers about the same manuscript you have reviewed. By comparing your comments with those of others, you could, indirectly, assess your own performance. Participating in the peer‐review process may help you learn to evaluate scientific evidence critically and enhance your professional development by improving research skills to improve the contribution to overall knowledge development.
